# Nanoparticle-Based Antioxidants in Stress Signaling and Programmed Cell Death in Breast Cancer Treatment

**DOI:** 10.3390/molecules28145305

**Published:** 2023-07-10

**Authors:** Yedi Herdiana, Sriwidodo Sriwidodo, Ferry Ferdiansyah Sofian, Gofarana Wilar, Ajeng Diantini

**Affiliations:** 1 Department of Pharmaceutics and Pharmaceutical Technology, Faculty of Pharmacy, Universitas Padjadjaran, Sumedang 45363, Indonesia; sriwidodo@unpad.ac.id; 2 Department of Pharmaceutical Biology, Faculty of Pharmacy, Universitas Padjadjaran, Sumedang 45363, Indonesia; ferry.ferdiansyah@unpad.ac.id; 3 Department of Pharmacology and Clinical Pharmacy, Faculty of Pharmacy, Universitas Padjadjaran, Sumedang 45363, Indonesia; g.wilar@unpad.ac.id (G.W.); ajeng.diantini@unpad.ac.id (A.D.)

**Keywords:** breast cancer, reactive oxygen species, DNA damage, inflammation, angiogenesis, cell death

## Abstract

Breast cancer (BC) is a complex and heterogeneous disease, and oxidative stress is a hallmark of BC. Oxidative stress is characterized by an imbalance between the production of reactive oxygen species (ROS) and antioxidant defense mechanisms. ROS has been implicated in BC development and progression by inducing DNA damage, inflammation, and angiogenesis. Antioxidants have been shown to scavenge ROS and protect cells from oxidative damage, thereby regulating signaling pathways involved in cell growth, survival, and death. Plants contain antioxidants like ascorbic acid, tocopherols, carotenoids, and flavonoids, which have been found to regulate stress signaling and PCD in BC. Combining different antioxidants has shown promise in enhancing the effectiveness of BC treatment. Antioxidant nanoparticles, when loaded with antioxidants, can effectively target breast cancer cells and enhance their cellular uptake. Notably, these nanoparticles have shown promising results in inducing PCD and sensitizing breast cancer cells to chemotherapy, even in cases where resistance is observed. This review aims to explore how nanotechnology can modulate stress signaling and PCD in breast cancer. By summarizing current research, it underscores the potential of nanotechnology in enhancing antioxidant properties for the treatment of breast cancer.

## 1. Introduction

Breast cancer (BC) is a multifaceted condition with varied molecular subtypes and outcomes, accounting for a substantial number of new diagnoses, estimated at around 2.3 million cases [1,2,3]. Insufficient nutrition, lack of physical activity, and exposure to pollutants associated with modern lifestyles increase the body’s production of free radicals, causing oxidative stress [4,5,6,7]. Oxidative stress and the generation of reactive oxygen species (ROS) are associated with cancer progression [1,8,9]. Oxidative stress is a hallmark of BC, where there is an inequilibrium between the generation of ROS and the body’s ability to counteract them through antioxidant defense mechanisms. ROS can induce DNA damage, inflammation, and angiogenesis, all of which contribute to the development and progression of BC [10,11]. Excessive oxidative stress can lead to various health problems including carcinogenesis, inflammation, aging, diabetes, and cardiovascular disease [1,9,12,13].

ROS and antioxidant molecules, in conjunction with electrical signaling, constitute an integral part of the cellular and organismal stress signaling network. These processes work together to control the expression of genes and allocate energy for growth, adaptation, or protection, ultimately impacting how cells remember and respond to stress and communicate with each other [14]. Tumor cells commonly exhibit elevated levels of ROS in comparison to healthy cells. They also exhibit a strong ability to regulate redox homeostasis to maintain a relatively low level of oxidative stress. This stands in contrast to the redox balance observed in normal cells [8,11]. ROS exhibit a complex role in cellular dynamics, exerting both promoting and inhibitory effects on cancer growth contingent upon specific circumstances. During the initial stages of cancer formation, ROS contribute to the development of cancer by promoting oxidative stress and introducing genetic mutations that disrupt the regulation of cell growth. As a tumor progresses, ROS facilitate cancer cell invasion of surrounding tissues and metastasis by activating specific signaling pathways [15]. The presence of ROS triggers oxidative stress, which impacts diverse biological processes involving PCD such as apoptosis, necrosis, and autophagy [16]. Emerging studies indicate that ROS play a crucial role by signaling molecules throughout the entirety of the cellular process leading to cell death. Excessive ROS production can harm cellular structures and molecules, triggering inflammation, which is a known factor in the development of diabetes and cancer [17]. PCD is a vital mechanism for maintaining tissue balance and plays a significant role in cancer progression [18]. When the control of apoptosis is disrupted, cancer cells can survive longer, accumulate more mutations, stimulate the formation of blood vessels, enhance cell proliferation, disturb differentiation, and increase invasiveness as the tumor progresses [19]. Cancer cells have a reduced ability to undergo PCD, which contributes to their resistance to therapy. However, chronic exposure to low levels of ROS can lead to a suppression of PCD and promote cell survival, which can contribute to cancer development and progression [20]. Inducing PCD in cancer cells is an important therapeutic strategy.

Antioxidants protect cells, regulate stress signaling, and promote PCD in BC [8,21]. Plant-derived antioxidants like ascorbic acid, tocopherols, carotenoids, and flavonoids show potential anticancer properties [22]. Combination therapy with different antioxidants can target multiple signaling pathways and overcome resistance [23]. Antioxidants should scavenge ROS, regulate cell signaling, induce PCD, minimize side effects, synergize with other anticancer agents, and be personalized for tumor characteristics. They also protect normal cells from chemotherapy side effects, but their impact on cancer cell efficacy varies. The existing approaches for preventing and treating cancer have several limitations including limited efficacy, significant toxicity, and high costs. Consequently, there is a critical need to explore and develop innovative multi-targeted agents that can effectively modulate abnormal signaling in cancer. The discovery and advancement of such agents carry immense importance in enhancing the outcomes of cancer treatment while concurrently reducing the occurrence of adverse effects and overall treatment expenses. Exogenous and endogenous antioxidants interact intracellularly and extracellularly, enhancing activity and influencing tumor microenvironments (TMEs). Some antioxidants regulate gene expression and antioxidant defense pathways [8,10,24].

Nanomaterials enable controlled drug delivery with adjustable size, surface charge, and morphology [25,26]. Nanoparticles enhance antioxidant bioavailability, stability, solubility, and tumor cell targeting [27,28,29]. Nanoparticles serve as efficient delivery vehicles for antioxidants, enhancing their antioxidant activities and overall effectiveness [30]. Functionalization with ligands or antibodies improves efficacy and reduces off-target effects [7,31,32]. Through the use of nanoparticles to encapsulate antioxidants, their effectiveness can be significantly improved, specifically targeting BC cells. Encouragingly, antioxidant-loaded nanoparticles have demonstrated notable success in triggering PCD and sensitizing BC cells to chemotherapy, effectively overcoming resistance challenges. The integration of nanotechnology into antioxidant delivery holds immense potential in advancing BC treatment strategies and improving patient outcomes [33,34,35,36]. Antioxidant combination therapy shows promise in regulating stress signaling and PCD in BC [37,38]. We summarize that antioxidants and their combination therapy hold promise for BC treatment. However, further research is required to overcome existing challenges and fully exploit the potential and future directions in the development of antioxidant-based therapies for BC.

## 2. Stress Signaling and Programmed Cell Death in BC

BC is divided into three main subtypes based on specific biomarkers: hormone-receptor-positive/ERBB2-negative, ERBB2-positive, and Triple Negative Breast Cancer (TNBC) [39]. Cancer occurs due to abnormal cell growth and uncontrolled cell death, which can spread to other tissues. Cancer research emphasizes studying not just cancerous cells themselves, but also the neighboring cells comprising a TME. A TME includes tumor cells, neighboring cells, and immune cells. The TME can display stress characteristics like genomic instability, hypoxia, and increased levels of ROS. ROS play a dualistic role in the progression of cancer, exhibiting both supportive and inhibitory effects on malignant behavior [8,10,40,41]. ROS and antioxidant molecules, working in coordination with electrical signaling, play vital roles in the stress signaling networks within cells and organisms [14].

### 2.1. Sources of ROS in BC

BC is a complex disease that arises due to various genetic, environmental, and lifestyle factors [42,43,44]. ROS have a substantial impact on the development and progression of BC [45,46]. The elevated levels of ROS in BC cells can contribute to genomic instability, tumorigenesis, invasion, and metastasis [47,48]. Moreover, ROS can activate various signaling pathways, including PI3K/AKT, MAPK/ERK, and NF-κB, that promote cell survival, proliferation, and angiogenesis [49,50].

An imbalance in redox homeostasis, characterized by an excessive presence of reactive oxygen species (ROS), is known to disrupt cellular equilibrium [51,52,53]. These highly reactive radicals originate from both internal sources, such as mitochondria and inflammatory cells, as well as external sources like environmental toxins, radiation, and various chemical compounds found in alcohol, tobacco smoke, and certain drugs. The presence of these free radicals can inflict harm upon essential biomolecules, including lipids, proteins, and nucleic acids, thereby inducing oxidative stress and causing damage to various human tissues [54,55]. Exposure to environmental pollutants like di(2-ethylhexyl) phthalate (DEHP), vanadium (V), Cu, and Cd can harm our health and cause oxidative damage in different organs [56,57,58]. For example, DEHP can injure the spleen, but lycopene may protect against it. V can cause mitochondrial problems, while Cu can lead to kidney damage through oxidative stress and autophagy. Cd exposure can impair lung function and mitochondria, but selenium can help mitigate these effects. In summary, these pollutants can cause harm by disrupting our body’s balance and increasing oxidative stress, but certain substances like lycopene and selenium may offer protection [58,59].

ROS accumulation in BC cells is influenced by different factors; in estrogen receptor-positive (ER+) BC cells, ROS production increases due to amplified mitochondrial biogenesis and oxidative phosphorylation. HER2/neu-positive BC cells generate more ROS through the activation of NADPH oxidase (NOX) enzymes. Hypoxia, common in solid tumors, stimulates ROS generation by stabilizing HIF-1α and increasing mitochondrial respiration. Chemotherapy resistance in BC cells is linked to higher ROS levels as cancer cells use ROS scavenging mechanisms to evade cell death. Not only tumor cells but also other components of the tumor microenvironment contribute to ROS production [47].

The protein Drp1, which is involved in dividing mitochondria, is found to be increased in breast tumors and lymph node metastases associated with Triple Negative Breast Cancer (TNBC). This mitochondrial division process contributes to cell migration and invasion. Cells with high invasive potential show higher rates of mitochondrial biogenesis, oxidative phosphorylation, and oxygen consumption compared to non-invasive cells. Moreover, different types of breast tumors and breast cancer cell lines with varying abilities to spread to other parts of the body have been observed to have different mitochondrial characteristics [60].

Cancer cells can regulate their antioxidant capacities to maintain a specific redox balance, allowing for disease progression and cell survival [61,62]. ROS can activate cancer-promoting pathways and affect tumor-suppressing mechanisms and cell death. Triple Negative Breast Cancer (TNBC) cells have higher ROS levels compared to non-cancerous or hormone receptor-positive breast cancer cells. Targeting ROS in breast cancer treatment holds promise. ROS contributes to breast cancer development, and studying it helps develop treatments that exploit cancer cells’ metabolic weaknesses. By disrupting ROS-related pathways, researchers aim to hinder cancer cell growth and survival, potentially leading to more effective and personalized treatments for breast cancer.

### 2.2. Molecular Pathways of Stress Signaling and PCD in BC

BC is a multifaceted condition marked by the accumulation of genetic and epigenetic alterations, enabling uncontrolled cell growth while impeding PCD [63,64,65,66]. Multiple PCD pathways, such as apoptosis, necroptosis, and pyroptosis, have been identified, employing distinct molecular and cellular mechanisms to achieve diverse outcomes [67]. These pathways play crucial roles in eliminating undesirable or damaged cells from the organism [68,69,70]. However, in BC, PCD becomes dysregulated, resulting in the survival and proliferation of cancer cells [71,72,73]. The process of PCD can be delineated into three stages: initiation, execution, and degradation and clearance. By elucidating the aberrations in PCD regulation, we can gain insights into the mechanisms underlying BC pathogenesis and potentially identify novel therapeutic targets [74].

During the initiation stage of PCD, various internal and external signals can trigger the apoptotic pathway, leading to the activation of initiator caspases. The initiator caspases then cleave and activate downstream effector caspases, which promote the dismantling of cellular components and ultimately result in cell death [20,67,75]. In BC, the initiation stage of PCD can be disrupted by various mechanisms such as mutations in tumor suppressor genes or the activation of survival signaling pathways [76].

In the execution stage of PCD, the effector caspases cleave various substrates, leading to the fragmentation of the cell nucleus and the dismantling of the cytoskeleton. This results in the characteristic morphological changes associated with apoptosis such as cell shrinkage and membrane blebbing [67,75]. In BC, the execution stage of PCD can also be disrupted by various mechanisms such as the overexpression of anti-apoptotic proteins or loss of pro-apoptotic proteins [77,78].

Finally, in the degradation and scavenging stage of PCD, the apoptotic cell is phagocytosed by neighboring cells or macrophages, preventing the release of potentially harmful cellular contents into the surrounding tissue [67,75]. In BC, the degradation and scavenging stage of PCD can be disrupted by various mechanisms such as the overexpression of anti-phagocytic signals or defects in the clearance of apoptotic cells. Understanding the dysregulation of PCD in BC is crucial for developing new therapeutic strategies that can selectively target cancer cells while sparing healthy cells [79,80].

The connection between PCD (apoptosis) and ROS in BC is intricate and multifaceted. Maintaining a delicate equilibrium of ROS is essential for regulating the dualistic nature of cellular responses. Moderate levels of ROS activate oncogenic signaling pathways through redox mechanisms. However, excessive levels of ROS, referred to as oxidative stress, can lead to detrimental effects such as DNA, protein, and lipid damage. ROS can be generated in either the mitochondria or cytosol through various mechanisms. Interestingly, the outcomes of redox systems can vary significantly depending on the specific cancer type, TME, stage of cancer progression, and metastasis [81]. ROS in breast cancer influence the TME by promoting inflammation, angiogenesis, and immune evasion. They stimulate the release of pro-inflammatory cytokines, facilitate the growth of new blood vessels, and suppress the immune response. These actions support tumor growth and metastasis in breast cancer [82,83]. Nevertheless, if the levels of ROS exceed a specific threshold, it can result in cellular harm and, intriguingly, initiate antitumorigenic signaling by inducing oxidative stress. This response to oxidative stress presents a potential target for cancer therapies [39].

### 2.3. Key Functions and Factors Regulating PCD in BC

PCD is a regulated type of cell death that can be influenced by different biomolecules. It differs from accidental cell death (ACD) [20] and includes various subtypes such as autophagy-dependent cell death, apoptosis, mitotic catastrophe, necroptosis, ferroptosis, pyroptosis, and anoikis [84]. Apoptosis, which is considered type I cell death, is a rapid process primarily controlled by the caspase proteolytic cascade. Autophagy, categorized as type II cell death, involves the breakdown of damaged proteins and dysfunctional organelles through the formation of autophagosomes. Necrosis, known as type III cell death, encompasses various cell death modalities like necroptosis and pyroptosis. Entosis, classified as type IV cell death, involves one cell engulfing another and has distinct cytological characteristics. Furthermore, there are other forms of cell death, including ferroptosis, parthanatos, necrotic cell death, lysosomal-dependent cell death, alkaliptosis, and oxeiptosis [85].

The dysregulation of PCD is a significant factor in the development of BC [86]. Numerous studies have revealed disruptions in the molecular mechanisms underlying apoptosis, which constitute the primary pathway of PCD in breast malignancies. In recent years, there has been a growing interest in investigating the potential and effectiveness of inducing cell death through necroptosis in various types of cancer including BC [87]. In the degradation and scavenging stage, cancer cells can prevent the clearance of apoptotic bodies or promote the release of pro-inflammatory factors, contributing to the progression of the disease [74].

Multiple factors have been discovered to regulate PCD in BC cells. Among these factors, the TME, consisting of the extracellular matrix, stromal cells, and immune cells, plays a crucial role. Depending on the specific type and stage of BC, the TME can either facilitate or hinder PCD. Xu et al. emphasize the interconnectedness between different forms of cell death and the intricate diversity and complexity of immune cell infiltration within the TME [88]. For instance, a hypoxic TME can activate hypoxia-inducible factor 1-alpha (HIF-1α), which in turn activates prosurvival pathways and inhibits PCD. On the other hand, immune cells such as natural killer cells and cytotoxic T cells can induce PCD in BC cells by releasing cytotoxic molecules such as perforin and granzyme B.

Resistance to apoptosis, a process known as one of the fundamental characteristics of cancer, is well-established. Apoptosis, a process of PCD, can be initiated through two main pathways: the intrinsic pathway, also known as the mitochondrial pathway, which is primarily activated by intracellular stress signals like oxidative stress, and the extrinsic pathway, also referred to as the death receptor pathway, which is triggered by external signals. The death receptor pathway is initiated when death ligands bind to death receptors located in the extracellular domain. Examples of such death receptors include Receptor 1/Tumor Necrosis Factor-α (TNFR1/TNF-α) and Fas Receptor/Fas Ligand (FasR/FasL) among others. By paraphrasing this information, we can say that cancer cells possess the ability to evade apoptosis, which is a characteristic feature of cancer. Apoptosis is regulated by two main pathways: the intrinsic pathway, which is activated by internal stress signals like oxidative stress, and the extrinsic pathway, which is initiated by external signals through the binding of death ligands to death receptors such as TNFR1/TNF-α and FasR/FasL [89].

Alterations in the dynamics and functionality of mitochondria have been linked to malignancy in various types of cancer. In the case of invasive BC cells, there is an increase in oxidative phosphorylation (OXPHOS), mitochondrial biogenesis, and oxygen consumption rates compared to non-invasive cells. Moreover, primary human breast tumors display variations in mitochondrial mass while BC cell lines with varying metastatic capabilities exhibit diverse mitochondrial functional characteristics. The basal TNBC subtype primarily relies on mitochondrial ROS production to sustain oncogenic signaling, making it a potential therapeutic target [60].

Epigenetic mechanisms play crucial roles in cancer biology, influencing tumor growth, invasion, and immune response within the TME. Targeting dysregulated epigenetic mechanisms with small molecule compounds can be feasible. Furthermore, modulating the epigenome in solid cancers sensitizes cancer cells to immune attacks, increasing their responsiveness to immunotherapy [90]. Epigenetic modifications, including DNA methylation, histone acetylation, and microRNA expression, also regulate PCD in BC [91]. These modifications can impact the expression of PCD-related genes and influence the sensitivity of BC cells to chemotherapy and radiation therapy. For instance, the hypermethylation of the promoter region of the death receptor 5 (DR5) gene has been associated with resistance to apoptosis induced by TRAIL in BC cells [92].

### 2.4. Dynamic Changes in ROS Levels during PCD

ROS play a crucial role in various biological processes, and their generation within normal cells is strictly regulated to maintain a delicate equilibrium between their advantageous and detrimental effects. Intracellular ROS levels can reflect cell redox status and be altered under pathological conditions via different post-translational modifications [56]. The TME is also subject to the dynamic influence of ROS, which contributes to angiogenesis, metastasis, and cell survival. In normal cells, the levels of ROS are meticulously controlled through detoxification mechanisms facilitated by antioxidant enzymes, ensuring the preservation of redox balance and cellular homeostasis [45]. However, in cancer, there is an upsurge in ROS production, leading to oxidative stress and the accumulation of substantial levels of ROS [51,52]. Consequently, cancer cell proliferation is stimulated, angiogenesis is promoted, and metabolic activity is increased. The generation of ROS can be initiated through both internal and external pathways, thereby triggering the activation of growth factors like vascular endothelial growth factor (VEGF) and the transcription factor HIF-1α. Ultimately, these events contribute to the metastasis of tumor cells and the progression of cancer [93].

Elevated levels of ROS in conjunction with the decreased expression of cellular antioxidant enzymes contribute to the progression of malignancy by impacting multiple molecular targets such as NF-κB and Nrf2. The activation of signaling pathways mediated by these crucial factors results in the creation of an inflammatory milieu, inhibiting PCD and fostering tumor growth, angiogenesis, and metastasis. These combined mechanisms facilitate the onset, advancement, and development of malignant tumors [45].

### 2.5. Cell Death Regulators

BC is characterized by uncontrolled cellular proliferation and impaired mechanisms of cell death. High levels of anti-apoptotic proteins such as Bcl-2 and Bcl-X(L) have been linked to resistance to cancer treatments, while the increased expression of pro-apoptotic protein Bax promotes cell death and enhances sensitivity to anticancer therapies. Moreover, Bax can influence the apoptotic process by interacting with apoptosis regulatory proteins like BAG-1 and heat shock proteins (Hsp70/Hsc70), which can impact the stress response and affect the function of various proteins involved in cell division and death [94]. Cell communication within the BC microenvironment occurs through direct and indirect mechanisms, and these communication patterns exhibit dynamic characteristics. A comprehensive understanding of the dynamic changes within the BC microenvironment is crucial for personalized diagnoses and treatment strategies for BC. External environmental factors and internal signaling molecules can interact with the localized microenvironment of BC [95].

Apoptosis, a form of PCD, occurs through two main pathways: the extrinsic pathway, triggered by the activation of death receptors, and the intrinsic pathway, which involves the participation of mitochondria. The activation of the extrinsic pathway is initiated by the binding of death ligands, such as tumor necrosis factor α (TNFα), Fas ligand (FasL), and TNF-associated apoptosis-inducing ligand (TRAIL), to their respective cell surface receptors, including TNF receptor 1 (TNFR1), Fas, and death receptor (DR) 4/5. This engagement of homologous death domains initiates the activation of the extrinsic pathway of apoptosis. This activation leads to the subsequent activation of caspase-8, initiating the terminal phase of apoptosis.

Another key regulator of cell death in BC is the tumor protein p53. p53 functions as a transcription factor that plays a crucial role in maintaining the stability of the genome by regulating processes such as DNA repair, cell cycle progression, and apoptosis. In healthy cells, the E3 ubiquitin ligase MDM2 tightly regulates the levels of p53 by facilitating its breakdown. However, in response to DNA damage, cellular stress, or oncogenic activation, p53 becomes stabilized and translocates to the nucleus, where it activates the transcription of genes involved in apoptosis and cell cycle arrest. In BC, mutations in the p53 gene are frequently observed, leading to the loss of its tumor suppressor function and promoting the development of cancer.

PCD is governed by specific signaling pathways and can be regulated by genetic signals or pharmacological interventions. Different subroutines of PCD have varying impacts on cancer development and the treatment response. In the early stages of the disease, cancer cells may exhibit resistance to anticancer treatments due to mutations that disrupt the PCD pathway, and the evasion of PCD is recognized as one of the significant characteristics of cancer. By simultaneously targeting multiple PCD signaling pathways through drug or gene interventions, it becomes possible to overcome drug resistance in cancer cells and achieve therapeutic goals. A comprehensive exploration of PCD in cancer and the potential treatment opportunities it presents has been extensively discussed by Peng et al. [20].

## 3. Molecular Mechanisms of Antioxidants in BC

The antioxidant system plays a vital role in cellular protection against oxidative stress, preserving the balance of redox reactions by counteracting the harmful effects of ROS that arise from regular metabolic processes or because of exposure to external stressors. This system comprises enzymatic and non-enzymatic antioxidants that work together to protect cellular macromolecules from indiscriminate oxidative damage. The antioxidant system is composed of exogenous and endogenous antioxidants to maintain homeostasis [96].

### 3.1. Enzymatic Antioxidants

Antioxidants serve as a defense mechanism against oxidative stress, working to eliminate reactive species and maintain a balanced redox state. Among the enzymatic antioxidants, catalase, glutathione peroxidase (GPX), and superoxide dismutase (SOD) are particularly significant [84].

1. Superoxide dismutases (SODs)

Superoxide dismutase (SOD) is an enzyme that plays a crucial role in protecting DNA, cell membrane molecules, and proteins from oxidative disturbances. SOD is classified into three types, each present in numerous organisms and possessing different cofactors: Cu/Zn SOD1, Mn SOD2, and extracellular Cu/Zn SOD3 [97]. In the context of BC, the role of SOD is complex. Various studies have indicated that SOD levels increase as cancer progresses and that its ability to reduce oxidative stress can inhibit cancer growth. The production of O_2_ and H_2_O_2_ triggers the induction of SOD and catalase (CAT) activity. Inflammatory cells’ enhanced superoxide dismutase activity leads to an elevation in hydrogen peroxide production [98]. The superoxide dismutase family holds significant physiological importance in mitigating the detrimental effects of ROS [99].

The overexpression of SOD has been shown to impede in vitro proliferation, clonogenic survival, and invasion of TNBC cell lines, partially through the suppression of heparanase-mediated cell surface proteoglycan fragmentation and by reducing the bioavailability of VEGF. Additionally, in experimental lung and spontaneous metastasis mouse models, SOD overexpression significantly inhibited tumor metastasis, further underscoring the role of these extracellular enzymes in suppressing tumor development [99].

2. Glutathione peroxidase (GPX)

Glutathione peroxidase (GPX) is a crucial enzymatic antioxidant that plays a significant role in cellular protection. It catalyzes the reduction of hydrogen peroxide and organic peroxides, which are byproducts of cell metabolism and can induce oxidative stress [100]. GPX utilizes glutathione (GSH) as a co-substrate, leading to the oxidation of GSH and the formation of glutathione disulfide (GSSG). Another enzyme, glutathione reductase, along with NADPH as the reducing agent, regenerates GSH from GSSG. This recycling process ensures the continuous functioning of GPX as an effective antioxidant within cells.

To maintain normal levels of ROS, a systematic biological detoxification process is employed. For instance, in preadipocytes, N-acetylcysteine (NAC) reduces adipogenesis by reducing ROS levels. The use of chemotherapy drugs may lead to a reduction in the levels of reduced glutathione and antioxidant enzymes like glutathione S-transferase (GST) and GPX. This depletion is likely due to an increase in oxidative stress that occurs as a result of chemotherapy treatment [101,102].

3. Catalases

Catalase is another important antioxidant enzyme that helps mitigate oxidative stress by converting cellular hydrogen peroxide into water and oxygen. It plays a crucial role in cellular defense against oxidative stress [103,104]. Deficiency or dysfunction of catalase is associated with various diseases such as diabetes mellitus, vitiligo, cardiovascular disease, Wilson’s disease, hypertension, anemia, dermatological disorders, Alzheimer’s disease, bipolar disorder, and schizophrenia [104]. Genetic polymorphisms and mutations in the CTT1 gene can contribute to alterations in cellular oxidative status and the development of diseases including cancer [62]. Catalase expression and activity are increased in certain BC cells, suggesting a potential role in enhancing cancer cell survival and proliferation. However, catalase also exhibits tumor-suppressive effects in BC by protecting cells from oxidative damage, preventing DNA damage, and mutations that can contribute to cancer development.

Chemotherapy can decrease the levels of catalase and superoxide dismutase (SOD) enzymes in BC patients, leading to oxidative stress and diminished antioxidant capacity. Strengthening the body’s antioxidant system through the use of antioxidant supplements and dietary enrichment with natural antioxidant agents is crucial [102]. Curcumin, for example, has shown an antiproliferative effect in breast carcinogenesis, which may be related to its impact on catalase activity and protection against oxidative stress [103].

Modifications of catalase or the use of nano enzymes that mimic catalase activity present promising therapeutic strategies to enhance the frequency of CD4+ and CD8+ effector T cells while reducing the infiltration of immunosuppressive cells in the TME [105].

### 3.2. Non-Enzymatic Antioxidants

The use of antioxidant supplements as health enhancers and their potential anticancer effects have been topics of ongoing discussion. This debate extends not only to individuals in good health but also to patients diagnosed with cancer [62,106]. Natural antioxidants, such as vitamins A, C, E, and various plant-based compounds, possess the ability to counteract excessive free radicals in cancer cells by acting as hydrogen donors, quenching singlet oxygen, and delaying oxidative reactions [62]. Antioxidants derived from dietary sources, particularly from plants, including carotenoids, flavonoids, phenols, and vitamins, have shown distinct effects in inhibiting different stages of cancer development. They can induce cell cycle arrest and promote cancer cell death. These antioxidants exert their anticancer effects through various mechanisms including influencing cell signaling, altering cell cycle progression, and modulating enzymatic activity [54]. Numerous preclinical studies have demonstrated the potential of terpenoids as therapeutic agents in the treatment of various cancers including BC. Terpenoids exhibit the ability to regulate multiple transcription factors and intracellular signaling pathways, thereby inhibiting the initiation and promotion of carcinogenesis, as well as tumor invasion, metastasis, and angiogenesis, while inducing apoptosis [107].

### 3.3. Nicotinamide Adenine Dinucleotide Phosphate (NADPH)

Nicotinamide adenine dinucleotide phosphate (NADPH) serves as a crucial electron donor and indispensable cofactor involved in the transfer and storage of reduction potential for numerous anabolic reactions. Cancer cells regulate NADPH homeostasis through different signaling pathways and metabolic enzymes, which undergo adaptive alterations [108].

NADPH plays a crucial role in regulating the increased levels of ROS then dividing cancer cells and safeguarding dihydrofolate reductase (DHFR) against degradation. Moreover, NADPH is involved in the synthesis of the oncometabolite D-2-hydroxyglutarate (D-2HG) [84]. Recent research has revealed several mechanisms by which cancer cells regulate NADPH production: (1) the activation of AKT phosphorylates NAD kinase (NADK), leading to increased activity; (2) the promotion, by mutant p53, of the enhanced synthesis of NADPH from NADP by upregulating glucose-6-phosphate dehydrogenase (G6PD); and (3) the interaction between calmodulin and NADK, which enhances NADPH production. These adaptations ensure an adequate supply of NADPH to meet the demands of replication and protect against ROS [109].

Functionally, NADPH plays a dual role in antioxidant defense. On one hand, NADPH is utilized by glutathione reductase to convert oxidized glutathione (GSSG) to reduced glutathione (GSH), which serves as a co-substrate for glutathione peroxidase (GPX). GPX then reduces hydrogen peroxide (H_2_O_2_) and other peroxides to water (H_2_O) or alcohol, thus neutralizing ROS. On the other hand, NADPH acts as an electron donor for thioredoxin reductase (TRXR), maintaining the reduced form of thioredoxin (TRX). This process contributes to the scavenging of H_2_O_2_ and provides reduced ribonucleotide reductase (RNR) for DNA synthesis. Moreover, in certain cell types, NADPH binds to catalase, an important enzyme responsible for the breakdown of H_2_O_2_, and reactivates it when it has been inactivated by H_2_O_2_ [110].

Cancer cells rely on three distinct pathways—the nicotinic acid (NA) pathway, the de novo pathway, and the NAD salvage pathway—to generate NAD levels. Mutations in enzymes involved in NAD and NADPH metabolism can lead to changes in gene expression through epigenetic modifications, affecting various cellular processes. The increased availability of NADPH provides cancer cells with robust biosynthetic capacity and protection against oxidative stress, allowing for rapid proliferation. Although targeting NADPH metabolism has shown promise in certain cancers, its clinical application is limited due to associated toxicity [109,110].

### 3.4. Nuclear Factor E2-Related Factor 2 (NRF2)

The abnormal metabolism observed in tumor cells is closely linked to the development of BC, and the expression of Nrf2 plays a significant role in this process. Nrf2 has been found to increase cellular sensitivity to oxidants and electrophiles [111]. Increased levels of Nrf2 have been linked to the stimulation of breast cancer cell proliferation and migration. The inhibition of Nrf2 and increased expression of Kelch-like ECH-associated protein 1 (Keap1) result in the reduced expression of glucose-6-phosphate dehydrogenase (G6PD) and transketolase in the pentose phosphate pathway. Conversely, the overexpression of Nrf2 and depletion of Keap1 lead to opposite effects [112]. Nrf2 serves as a key regulator of antioxidant and cytoprotective systems. It accumulates in the nuclei of cells, where it forms heterodimers with small Maf proteins, and activates target genes by binding to specific regions called antioxidant response elements (ARE) or electrophile response elements (EpRE). This activation promotes cellular protection and defense mechanisms against harmful substances [111,113].

In healthy cells, Nrf2 expression is relatively low, maintaining redox homeostasis. However, cancer cells often exhibit the overexpression of Nrf2, leading to various phenomena such as drug resistance, angiogenesis, the development of cancer stem cells, and metastasis. Aberrant Nrf2 expression reduces the efficacy of therapeutic anticancer drugs and provides cytoprotection to cancer cells [114]. Nrf2 plays a crucial role in maintaining the delicate balance of cellular redox homeostasis in both normal and cancerous cells. The absence of Nrf2 leads to an upsurge in reactive oxygen species (ROS) production, which in turn causes DNA damage and promotes tumor formation. Nrf2 directly regulates the expression of vital molecules involved in antioxidant defense, including glutathione (GSH), thioredoxin (TXN), and NADPH, effectively controlling the levels of ROS within the cell. Interestingly, during oxidative stress, Nrf2 can be activated not only by NADPH but also by ROS generated through NADPH oxidase. The constitutive activation of the transcription factor NRF2, encoded by NFE2L2, has been observed in metastatic cells and tumors, resulting in the upregulation of a specific subset of NRF2-regulated genes. Depleting NRF2 results in elevated basal levels of ROS and significantly impairs the ability to develop primary tumors and form lung metastases [115].

Therefore, gaining a better understanding of cellular events and signaling cascades is crucial for developing effective therapeutic strategies against BC [114]. Targeting NRF2, a regulator of protective genes involved in antioxidant and anti-inflammatory responses, holds promise as a potential strategy for the prevention and treatment of cancer [88].

## 4. Antioxidant Delivery Systems: Current and Challenge

### 4.1. Antioxidants in BC

Antioxidants, commonly used as dietary supplements and in disease prevention, hold promising potential as adjuncts in cancer therapy by reducing treatment-related toxicity and improving patient outcomes [62,116]. They have been shown to enhance therapeutic efficiency and reduce the morbidity associated with radiotherapy and chemotherapy-induced toxicity, ultimately improving survival rates in cancer patients [17,18]. Under normal conditions, free radicals, including ROS, gradually accumulate in the human body, and their levels determine whether they trigger cell death or carcinogenesis [117,118]. Excessive damage caused by reactive oxygen species (ROS) can disrupt the permeability of mitochondrial membranes, resulting in the release of cytochrome C and triggering apoptotic cell death. To evade cell death, cancer cells exploit anti-apoptotic mechanisms like nuclear factor kappa B (NFκB), which is involved in enhancing the activated B cell pathway. Imbalances in cellular redox status activate redox-sensitive transcription factors such as nuclear factor 2 erythroid related factor 2 (Nrf2), NFκB, and activator protein 1 (AP-1) [119,120,121].

The combination of chemotherapy with cisplatin causes a decrease in plasma antioxidant levels. This decrease may indicate both a decrease in antioxidant levels that is possibly due to antioxidant consumption triggered by oxidative stress caused by chemotherapy as well as the loss of water-soluble antioxidants with small molecular weights, such as uric acid, through the kidneys [122]. Numerous studies have reported decreases in chemotherapy and radiotherapy toxicity with antioxidant supplementation [123]. For instance, the intake of multi-vitamins after a BC diagnosis has been associated with reduced mortality and recurrence rates. By strengthening the antioxidant defense system, antioxidants effectively alleviate these treatment side effects [124]. Furthermore, in addition to conventional cancer treatments like chemotherapy, radiotherapy, and surgery, natural products with antioxidant properties have shown promise in cancer prevention and treatment [123].

Flavonoids, a class of natural products found in many plants, can be classified into different groups based on their chemical structures. In low doses, flavonoids effectively eliminate the accumulation of free radicals and other oxidants during normal cellular processes. However, high doses of flavonoids, typically above the IC_50_ threshold, can induce severe oxidative stress in cancer cells and elevate ROS levels [39].

Natural products, such as phenolics, flavonoids, and carotenoids, have demonstrated efficacy in suppressing early and late-stage cancer development [125,126,127]. They accomplish this by focusing on early signals like DNA damage, oxidative imbalance, and cellular stress [39,128]. Epigenetic agents, whether used alone or in combination with traditional cancer drugs, present a promising approach for progress in this field [129].

While antioxidants have shown potential in cancer therapy, it is important to consider the potential health risks associated with high-dose antioxidant supplementation [130]. Studies have indicated that the excessive intake of certain antioxidants, such as beta-carotene and vitamin E, may increase the risk of specific cancers [131,132].

In the context of natural products with antioxidant properties, targeting the Nrf2 pathway has emerged as a potential anticancer approach. Nrf2, which is overexpressed in various human cancers, can be exploited by tumor cells for their survival. The activation of the Nrf2 pathway is important for cancer prevention; however, when control is lost, it can lead to detrimental effects such as accelerated cancer cell growth, evasion of senescence and apoptosis, and resistance to chemotherapy and radiotherapy [133].

The effectiveness of antioxidant therapy may depend on the level of ROS in cancer cells. In TNBC, which is characterized by high ROS levels, targeting this pro-oxidant state with antioxidants may hold promise. However, considering the variation among different tumor types and subtypes, more comprehensive analyses are necessary before considering antioxidant treatment as a universal therapy for cancer [60].

The daily diet serves as the primary source of natural antioxidant compounds, which can interact with each other and exert their antioxidant activity in a synergistic, additive, or antagonistic manner. When consumed, food undergoes gastrointestinal digestion, which can impact its antioxidant potential [134]. Our current knowledge regarding the pharmacokinetics and potential drug interactions between chemotherapy agents and self-administered adjuvants remains limited [135]. This knowledge gap arises from the diverse nature of antioxidant supplements and drugs, which lack comprehensive characterization in terms of pharmacological parameters and potential interactions with other medications. Consequently, it remains uncertain whether any intervention could adversely affect current patient therapy [124]. Cysteine depletion can trigger iron-dependent nonapoptotic cell death–ferroptosis [136,137]. Through the use of cysteine, which is involved in the synthesis of glutathione and coenzyme A, researchers discovered that targeting the system xC-subunit Slc7a11 induced tumor-selective ferroptosis and inhibited pancreatic ductal adenocarcinoma (PDAC) growth in genetically engineered mice. This effect was replicated by administering cyst(e)inase, a drug that depletes cysteine/cystine, providing a promising method to induce ferroptosis in PDAC [138]. For a comprehensive exploration of the use of antioxidants in BC, Carmen Griñan-Lison et al. provide an in-depth description [127]. Phytochemical-based nanocarriers offer a solution to the drawbacks of conventional breast cancer management. This article highlights their development for targeted drug delivery, aiming to improve patients’ quality of life and the healthcare system [139].

### 4.2. Combination Antioxidants

Combining various polyphenolic compounds can result in a synergistic effect in the treatment of cancer. These complex interactions can modulate cell physiology and signaling pathways, influencing the efficacy of drugs. However, polyphenols exhibit dual effects as both antioxidants and pro-oxidants, representing a “double-edged sword” in their potential therapeutic use. The combination of polyphenolic compounds with anti-tumor effects, particularly on BC cells, presents an intriguing avenue for further research, with a focus on understanding the individual components involved [140].

Studies have demonstrated synergistic effects between antioxidants and other drugs, as exemplified by the following findings:

- Flavanones exhibit remarkable antioxidant properties by effectively reducing the release of reactive oxygen species (ROS), formation of carbonylated proteins and lipid peroxides, and oxidation of reduced glutathione (GSH) to its oxidized form (GSSG) in Caco-2 cells. Moreover, these compounds demonstrate notable anti-inflammatory effects by inhibiting cyclooxygenase (COX) enzymes.
- Lavanones demonstrate significant antioxidant properties by reducing the release of ROS, formation of carbonylated proteins and lipid peroxides, and oxidation of GSH to GSSG in Caco-2 cells. Additionally, they exhibit notable anti-inflammatory effects by inhibiting COX enzymes [141].
- Formulations containing antioxidants and energy supplies have shown effectiveness in treating sperm changes and significantly improving fertilization capacity [142].
- Combining NFAT inhibition with antioxidants like N-Acetylcysteine may offer benefits in the treatment and/or prevention of hearing loss [143].
- Antioxidants such as curcumin and oxadiazole demonstrate anti-schistosomal activity against adult worms, leading to severe morphological changes and death [144].
- The combination of ethanol extracts from basil leaves and binahong leaves exhibits a significantly strong antioxidant activity compared to that of each individual extract [145].

The combination of polyphenolic compounds can yield synergistic effects in cancer treatment [136].

### 4.3. Interaction—Antioxidants with Anticancers

Dietary supplements containing antioxidants and essential nutrients such as folic acid, vitamin C, and pyridoxine are known to counteract the excessive production of ROS and oxidative stress caused by chemotherapy. These supplements can help prevent vitamin deficiencies and mitigate other health issues in cancer patients [127,135]. Numerous drugs have been developed using the redox platform to target various oxidative stress pathways, as all types of cytotoxic cancer drugs can induce direct or indirect oxidative stress. While concerns have been raised that antioxidant supplementation may reduce the effectiveness of radiotherapy or chemotherapy, most studies indicate a reduction in side effects when supplements are used during these treatments [8]. Some data suggest that antioxidants may also protect normal tissues from chemotherapy-induced damage without compromising tumor control [24].

In a rat study, vitamin C demonstrated clear benefits in mitigating DOX-induced liver toxicity while vitamin E was effective in reducing DOX-induced kidney toxicity [146,147]. High-dose vitamin C has shown promise as an anticancer drug for BC, exhibiting selective toxicity toward cancer cells. This effect may be attributed to three potential mechanisms: the reaction of ascorbate with labile iron in the TME, resulting in the production of H_2_O_2_ and •OH; the diffusion of extracellular H_2_O_2_ into cancer cells to react with intracellular labile iron; and the contribution of extracellular H_2_O_2_ to increased extracellular DHA levels. BC cells have higher requirements for labile Fe^2+^ for survival and growth, and they reprogram iron metabolism through various mechanisms. Intracellular labile Fe^2+^ levels are higher in BC cells compared to normal breast epithelial cells, and advanced BC patients exhibit significantly higher plasma Fe^2+^ levels than healthy individuals [148,149].

Natural antioxidant vitamin C, when used at high doses, can enhance the efficacy of pharmacological therapy and/or reduce side effects in BC patients [150]. The combination of anti-cancer drugs with high doses of vitamin C has shown stronger inhibition of BC cell proliferation compared to using anti-cancer drugs alone. Furthermore, high doses of vitamin C have also demonstrated inhibitory effects on the growth of tamoxifen, doxorubicin, and docetaxel-resistant MCF-7 cells [151].

Coenzyme Q10 (CoQ10) and vitamin C treatment have been found to inhibit doxorubicin (Dox)-induced gastric mucosal injury by suppressing the activation of the IkKB/IκBα/NF-κB/p65/TNF-α pathway. This treatment promotes anti-inflammatory effects on gastric tissue and regulates the composition of intestinal flora [111]. Using nanocarriers for multi-target therapy can improve the effectiveness of anti-tumor treatment while minimizing side effects by enabling lower dosages. Combining natural products with gene therapy provides more benefits compared to using a single-treatment approach. Delivering multiple therapeutic agents or small interfering RNA (siRNA), which is a powerful gene editing tool, using nanocarriers can enhance their synergistic effects against tumor cells in cancer therapy [152].

Characterization studies have demonstrated favorable biopharmaceutical properties of nanocarriers such as small and uniform particle size, relatively high drug-loading capacity, good colloidal stability, and controlled drug release. In the case of doxorubicin, chitosan-polyethylene glycol-glycyrrhetinic acid nanoparticles (CPMSD) maintained the anticancer efficacy and mechanism of action, which were unaffected by the co-administration of synergistic antioxidant agents (SAA). Moreover, CPMSD exhibited systemic safety and cardioprotective effects against DOX-associated oxidative stress injuries in tumor-bearing mice [153].

### 4.4. Nanotechnology for Delivering Antioxidants

Nanotechnology-based drug delivery systems show promise in cancer prevention, diagnosis, and treatment. These systems encapsulate or conjugate drugs with different solubility profiles into biocompatible and biodegradable nanocarriers. Overcoming biological barriers and achieving targeted drug delivery are significant challenges in cancer therapy. Nanocarriers can target tumors through both passive targeting, using the enhanced permeability and retention (EPR) effects, as well as active targeting using site-specific ligands [29].

Lipid-based nanocarriers are widely used to improve the bioavailability, targeting efficiency, and delivery of therapeutic molecules. They offer advantages such as low toxicity, scalability, strong biocompatibility, and high drug-loading efficiency [154]. Due to their favorable surface-to-mass ratio, lipid-based nanocarriers exhibit enhanced uptake in the testis through mechanisms involving solubilization in the intestinal environment, intestinal lymphatic transport, and enterocyte-mediated transport [155]. Lipid-based nanostructures, including emulsions, liposomes, niosomes, solid-lipid nanoparticles (SLNs), and nanostructured lipid carriers (NLCs), are commonly used in drug delivery. Liposomes and niosomes are vesicles with aqueous cores while emulsions consist of lipid droplets stabilized by surfactants. SLNs have dense lipid cores and NLCs contain liquid lipid droplets within tightly-packed cores. Liposomes are versatile in encapsulating both hydrophilic and hydrophobic drugs, with hydrophilic molecules being loaded into the inner cavity and hydrophobic drugs being incorporated into the lipid bilayer [154].

Polymer-based nanostructures are frequently employed to manipulate biodistribution and enhance the natural antioxidant properties of nanocarriers. The biodistribution of nano-encapsulated bioactive compounds primarily depends on the sizes, shapes, chemical compositions, and surface properties of the encapsulated nanoparticles, making them less reliant on the physicochemical properties of the encapsulated active pharmaceutical ingredients [156]. Other types of nanoparticles, such as dendrimers and mesoporous silica nanoparticles, have shown potential for controlled antioxidant delivery. To facilitate the translation of this technology into practical antioxidant therapy, regulatory considerations and manufacturing processes need to be taken into account.

ROS generated by abiotic stresses cause damage and diseases, but the use of antioxidant-functionalized nanoparticles derived from biological sources offers a promising solution to combat oxidative stress [157]. The characterization results have revealed that silver nanoparticles synthesized using C. nocturnum extract exhibited higher antioxidant activity compared to vitamin C [158]. During the design process, nanocarriers can be tailored to encapsulate multiple drugs or genes. Implementing ncRNA-based therapy for targeting PCD may hold promise as a therapeutic strategy for BC [86]. Various small molecule compounds that target different PCD subroutines have been developed to improve cancer treatment. These include single, dual, or multi-target small molecule compounds, drug combinations, and emerging therapeutic strategies, all of which offer new directions to exploit the vulnerability of cancer cells through small molecule drugs targeting PCD for therapeutic purposes [20].

Multidrug-loaded nanocarriers offer the advantage of the simultaneous release of multiple therapeutic agents. These nanocarriers minimize drug–drug interactions and can improve the pharmacokinetic profile [159]. Pharmacodynamic analysis has demonstrated a synergistic effect when combining two drugs, Gef and Qur, within a single nanoparticle carrier. Encapsulation within the nanocarrier protects against degradation and enhances the solubility and absorption of polyphenols in the body. Furthermore, drug delivery systems (DDSs) enable targeted delivery to specific tissues or cells and allow for controlled release, leading to a sustained therapeutic effect [159]. Various nanocarriers are capable of the co-delivery of hydrophobic and hydrophilic antioxidants [152]. These surface active agents can also be combined with antioxidants to enable higher emulsification and better antioxidant properties [160].

## 5. Perspectives

BC poses a significant global health challenge, greatly affecting survival rates and the quality of life. Therefore, it is vital to evaluate the effectiveness of current treatments to reduce the burden it imposes [124]. Antioxidants have potential in preventing and treating neurodegenerative diseases including cancer, but their efficacy depends on the time of day, dosage, and specific conditions [161]. However, many natural antioxidants have low bioavailability, such as polyphenols, which are found in low concentrations in the blood [123,162,163]. As a result, the exact effect of the total antioxidant dose from food and supplements is still unclear [9]. Determining the exact effect of antioxidants from dietary supplements is challenging due to variations in manufacturer, age, and collection [135].

Cancer treatment costs have had a significant impact on limited access, emphasizing the need for more affordable options [164]. Antioxidants have emerged as promising agents for cancer prevention due to their availability, affordability, and broad effects [165,166,167]. However, the medical community holds divergent opinions on their use, particularly in conjunction with chemotherapy, as antioxidants may inadvertently protect both healthy and cancer cells from oxidative damage caused by chemotherapy drugs [168]. Often, individuals self-administer dietary supplements and vitamins during conventional cancer treatment without seeking professional advice [135]. Although generally considered safe, further research is necessary to determine the optimal combination and dosage of antioxidants for different types of cancer. Controlled trials focusing on specific regimens and cancer types can assess the safety and efficacy of antioxidant therapy [169,170,171]. Additionally, ongoing research aims to identify new biomarkers to evaluate treatment effectiveness.

Targeting ROS specifically in tumor cells presents a challenge in cancer treatment [172,173,174]. However, expanding knowledge about ROS can enhance our understanding of cancer and lead to advancements in ROS-based therapeutics. It is important to note that mitochondria-generated ROS can have both beneficial and detrimental effects [49]. Elevated ROS levels in cancer cells and the TME contribute to chronic inflammation and immune tolerance. Promisingly, combination therapy involving ROS-modulating agents and immunotherapy has shown potential in this regard. By deepening our understanding of the distinct redox signaling pathways in cancer cells, we can develop safe and effective therapies [8,174]. Manipulating the redox balance of cancer cells also holds promise for eliminating cancer stem cells and overcoming drug resistance [61,100,128]. Stress signaling and PCD are pivotal in maintaining overall health [71,175].

Nrf2, a tightly regulated transcription factor, is often exploited by cancer cells to defend against disruptions in the intra-cellular antioxidant/pro-oxidant balance caused by endogenous and environmental agents [176]. Although the activation of this pathway is crucial for cancer prevention, its loss of control can lead to detrimental effects such as rapid cancer cell growth, resistance to apoptosis, chemotherapy, and radiotherapy. To determine the optimal therapeutic approach, it is important to carefully evaluate the interplay between antioxidants, specific cancer types, the Nrf2 pathway, and ROS levels in cancer cells.

Nanocarriers have emerged as a widely employed strategy to enhance the bioavailability, targeting, and delivery efficiency of therapeutic molecules including antioxidants. However, clinical trials investigating antioxidant nanoformulations remain limited, and further extensive preclinical investigations are necessary to develop efficient and safe nano drugs for targeted delivery. Nanoantioxidants have demonstrated remarkable efficacy in mitigating oxidative stress by exhibiting enhanced sensitivity, cellular antioxidant activity, minimal cytotoxicity, and precise targeted delivery. The use of nanotechnology-based platforms shows promise in overcoming apoptosis resistance and enhancing tumor immunity [177,178]. By addressing these aspects, uncertainties surrounding the utilization of antioxidants as adjuvants in cancer therapy can be elucidated. Advancing the field of nanoantioxidants requires both the development of novel and effective therapeutic delivery systems as well as the design of reliable antioxidant activity assays for accurate measurement. These prerequisites are crucial in furthering our understanding and application of nanoantioxidants in therapeutic interventions.

## 6. Conclusions

The role of antioxidants is crucial in preserving the equilibrium of cellular redox balance by counteracting reactive oxygen species (ROS) and preventing oxidative damage. Their ability to counteract oxidative stress helps protect cells from injury and can influence cell death pathways, thereby contributing to overall cellular health and disease prevention. Nanotechnology can help improve solubility, bioavailability, and stability and reduce the potential toxicity associated with the use of antioxidants in cancer therapy. By gaining a better understanding of the mechanisms of stress signaling and PCD, as well as by further developing nanostructures, the use of antioxidant nanoparticles will shape a better future in the treatment of BC.
